# Supplementary figures and images for: Computational tools to detect signatures of mutational processes in DNA from tumours: A review and empirical comparison of performance
Source: PLoS One. 2019 Sep 12;14(9):e0221235. doi: 10.1371/journal.pone.0221235 (PMC6741849; doi:10.1371/journal.pone.0221235)

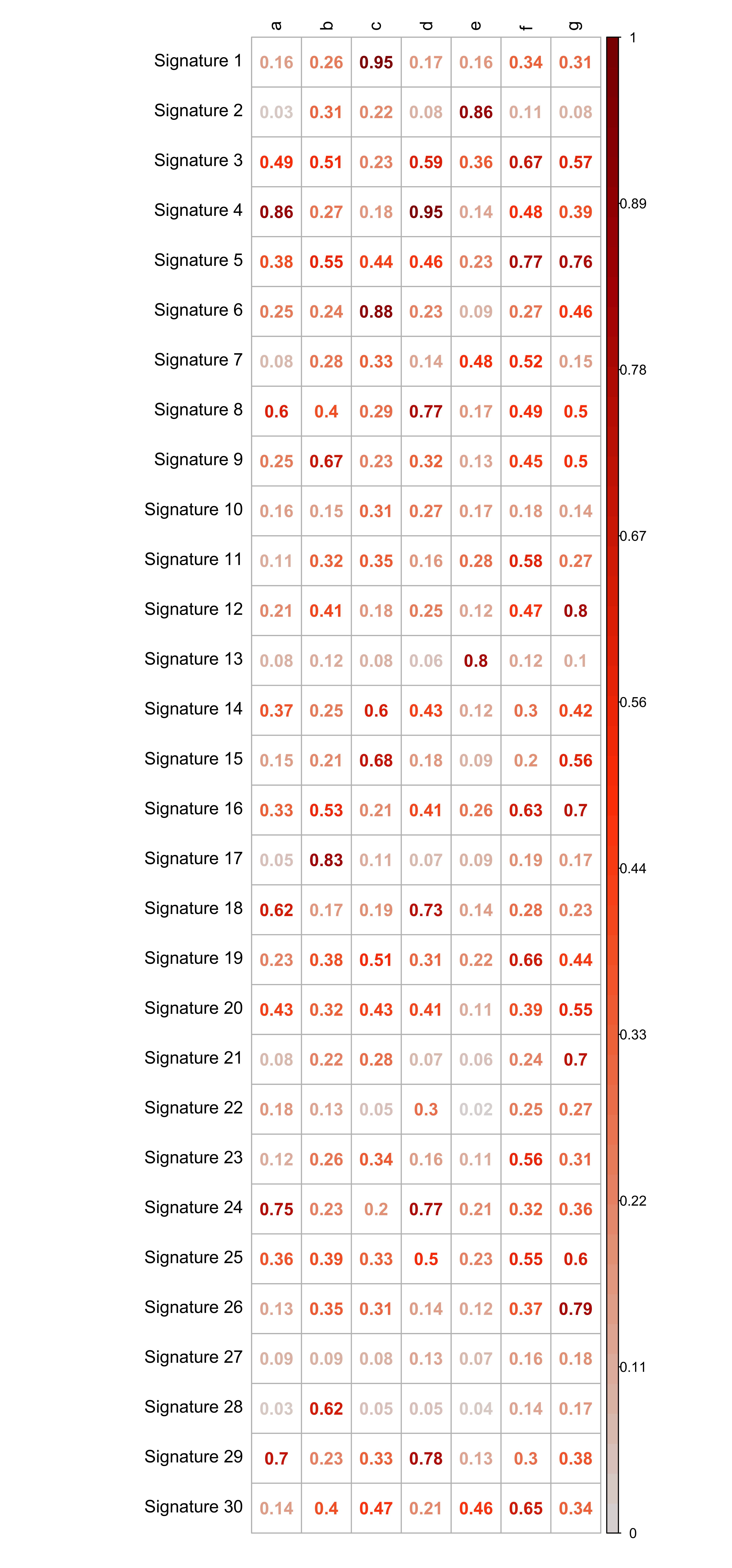

Supplement: S1 Fig — Signatures a-g were identified in a de novo extraction using the maftools R package from the TCGA Lung Adenocarcinoma cohort of 563 cancer genomes. The novel signatures were then compared to the 30 signatures validated in the COSMIC database in terms of cosine similarity. Each signature is then assigned to the most similar COSMIC signature provided that their cosine similarity is above a fixed threshold. For instance, signature f is matched to signature 5 at a cut-off of 0.75 but is considered as a completely new signature if the cut-off is at 0.80. Also note that a unique assignment can be controversial: for instance, signature g is similar both to signatures 12 and 26. (TIFF) [file pone.0221235.s001.tiff]

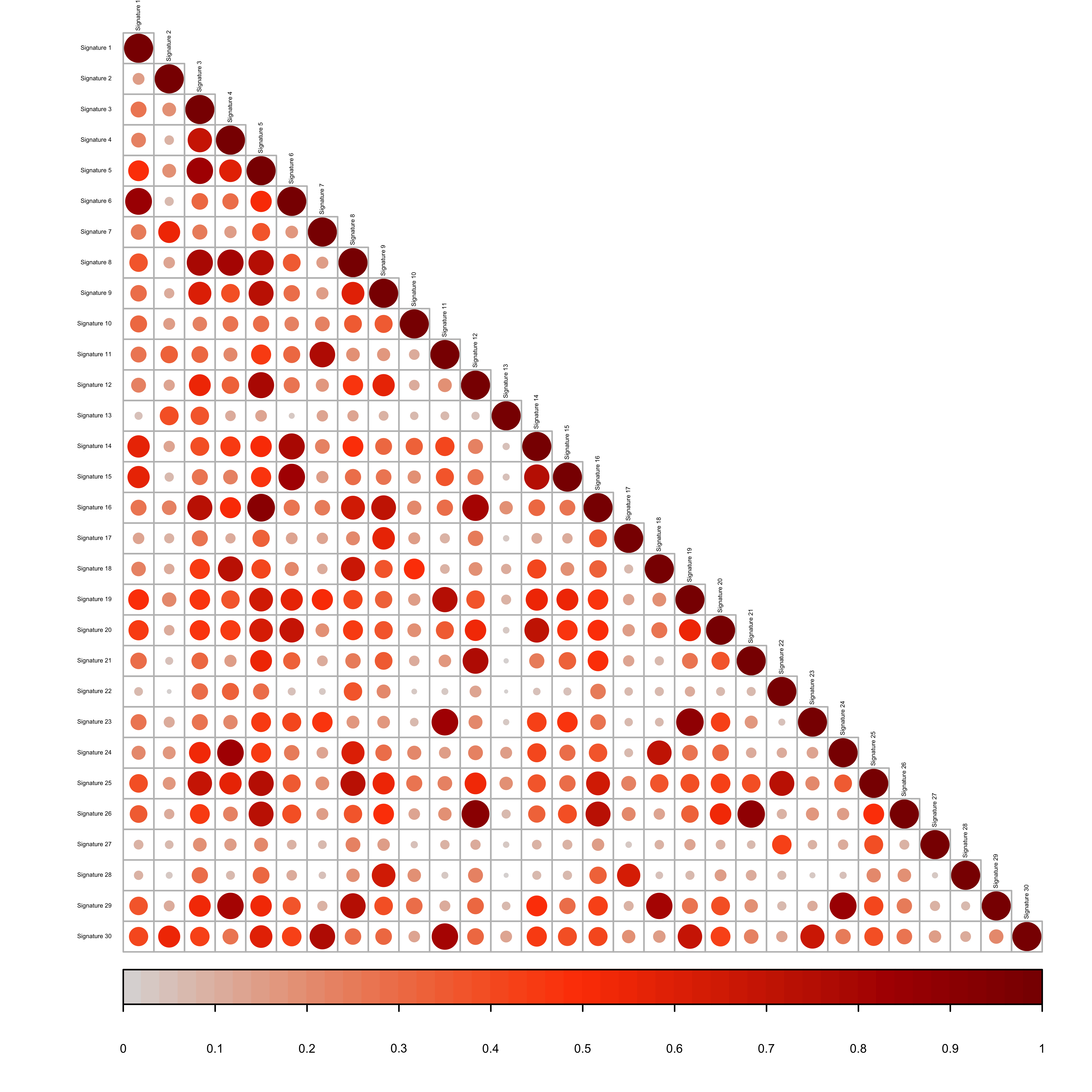

Supplement: S2 Fig — Some COSMIC signatures are very similar to others. For instance, signature 8 is similar to signatures 3, 4 and 5. (TIFF) [file pone.0221235.s002.tiff]

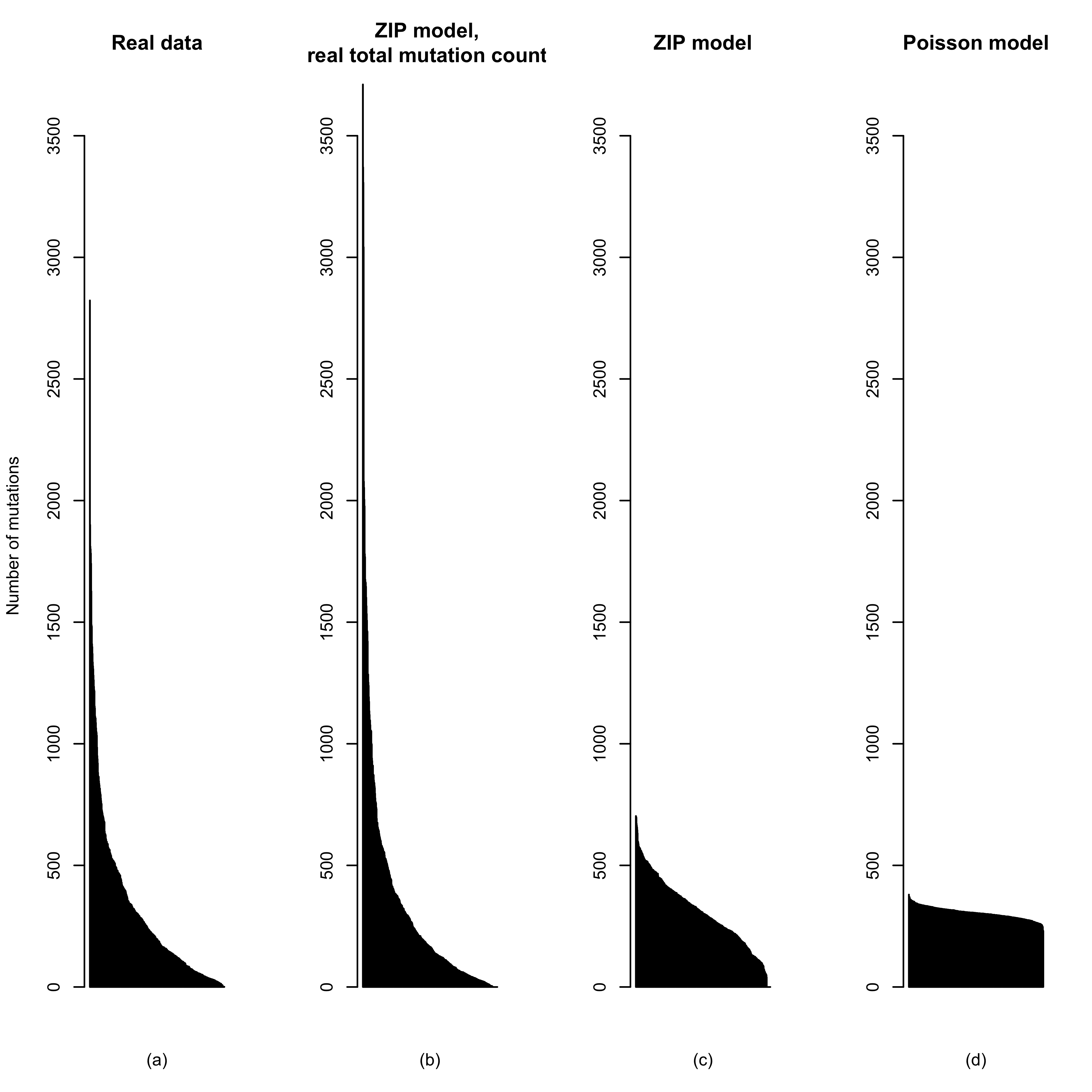

Supplement: S3 Fig — Part a: Real catalogues from the TCGA lung adenocarcinoma cohort. Parts b and c: Catalogues sampled from the ZIP model described in the main text. The relative contribution qn of each signature n is the mean of the relative contributions of n in all samples as estimated by maftools. In part b simulated and real catalogues are in a 1 to 1 correspondence: for each simulated sample g, the total number of mutations rg in the corresponding real catalogue is taken. In part c all samples are simulated according to r = 306, the average total number of mutations in the real data. The latter example illustrates the parametric model used for the simulation study. Part d: Catalogues sampled according to the Poisson model egn∼P(λn), where λn is the mean number of mutations due do to n in the real samples as estimated by maftools. (TIFF) [file pone.0221235.s003.tiff]

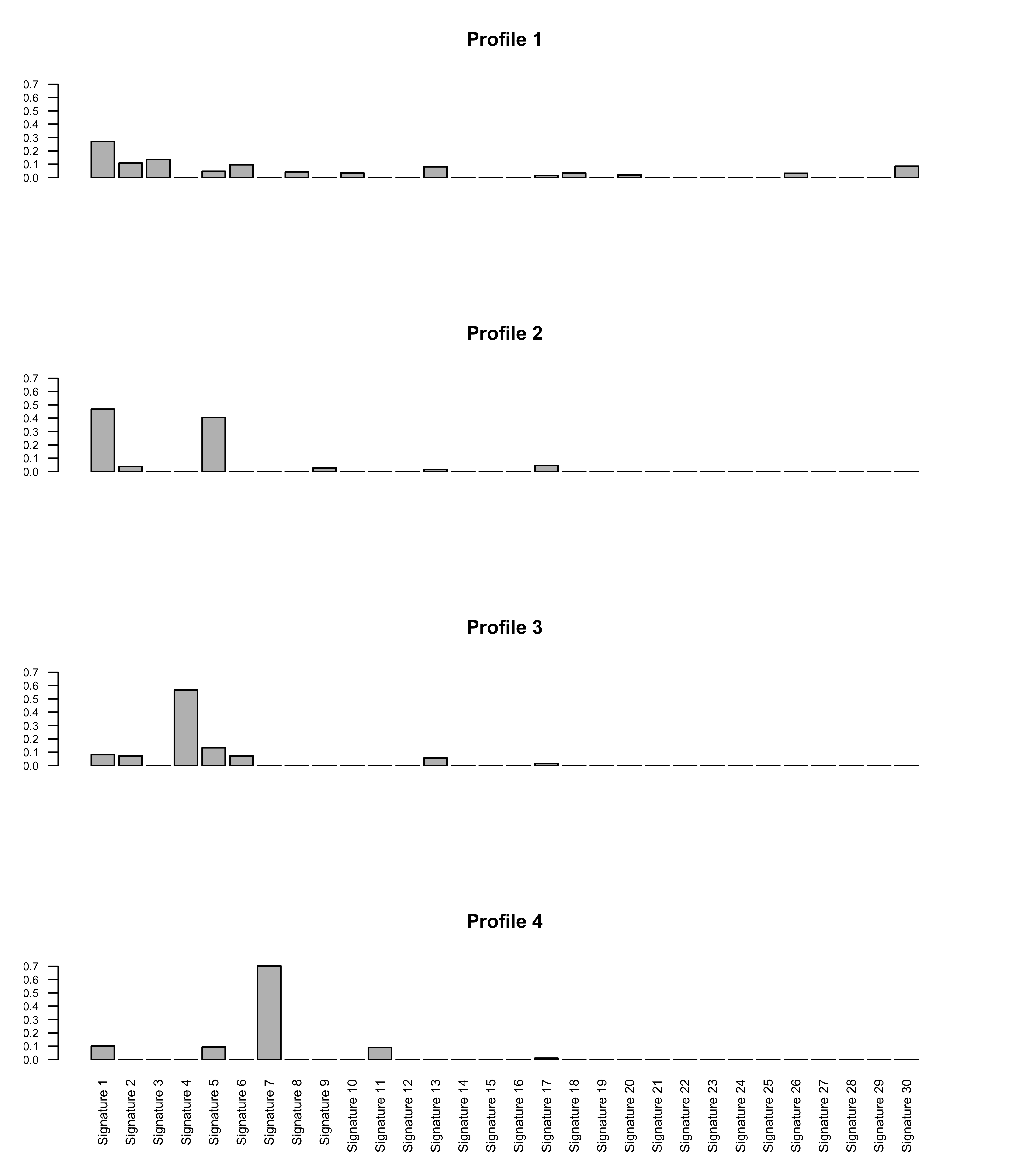

Supplement: S4 Fig — Four different configurations (q1,…,q30) were considered for simulating realistic data. Each configuration represents the average share of mutations due to the different COSMIC signatures and was chosen to mimic real exposure profiles for four cancer types: estimates were obtained from Breast Cancer (Profile 1), Lymphoma (Profile 2), Lung Adenocarcinoma (Profile 3) and Melanoma (Profile 4) TCGA cohorts. (TIFF) [file pone.0221235.s004.tiff]

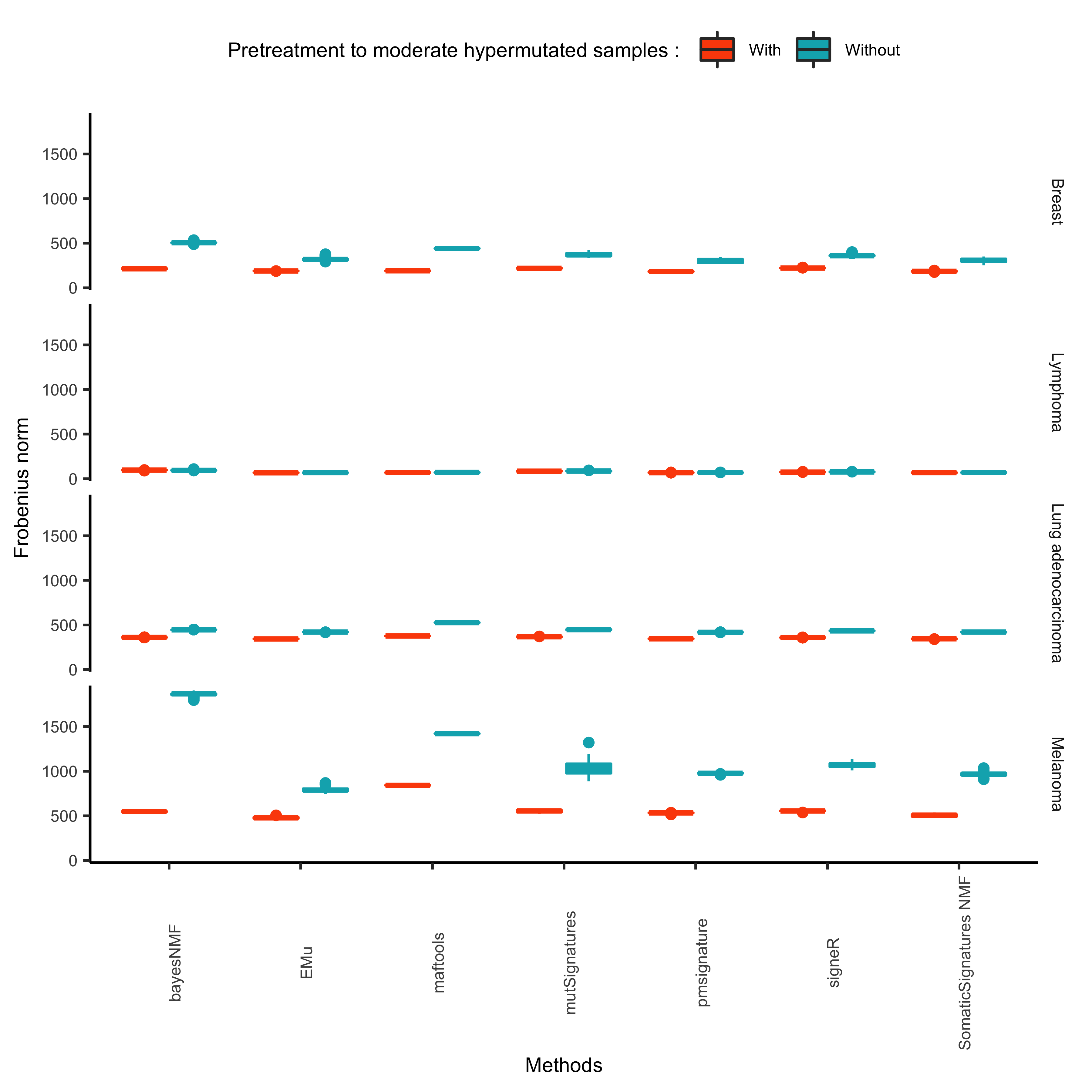

Supplement: S5 Fig — Each dataset was analysed with or without data pre-treatment with the bayesNMF get.lego96.hyper function. The profiles of the pre-treated catalogues are shown in S6 Fig. (TIFF) [file pone.0221235.s005.tiff]

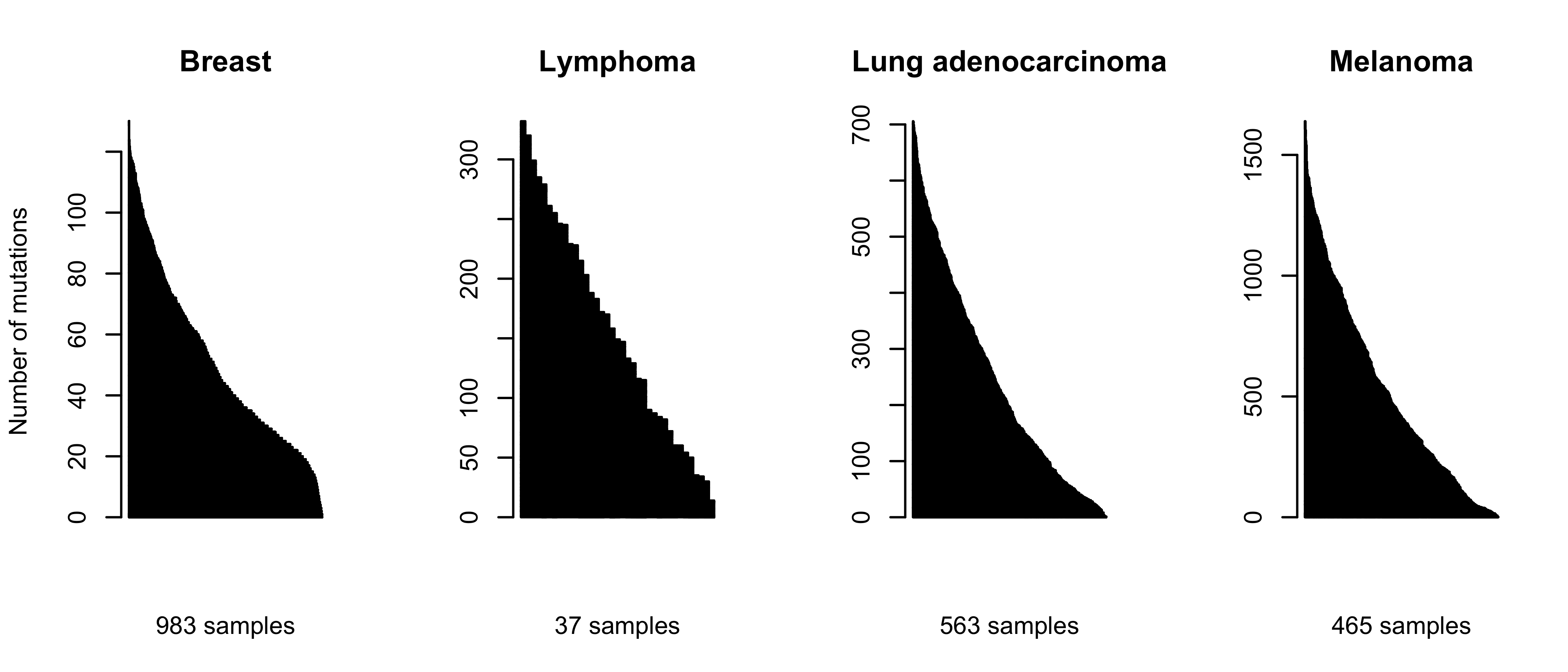

Supplement: S6 Fig — Datasets represented in Fig 1 were pre-treated using the bayesNMF function get.lego96.hyper. (TIFF) [file pone.0221235.s006.tiff]
