# Supplementary material for: Computational tools to detect signatures of mutational processes in DNA from tumours: A review and empirical comparison of performance
Source: PLoS One. 2019 Sep 12;14(9):e0221235. doi: 10.1371/journal.pone.0221235 (PMC6741849; doi:10.1371/journal.pone.0221235)
Supplement: S1 File — Description of our original method for signature refitting and the corresponding R script. (DOCX) [file pone.0221235.s007.docx]

MutationalCone

We report here the R code implementing our original method for signature refitting.

Let $S$ be the linear subspace of $\mathbb{R}^{K}$ spanned by the reference signatures. Our function MutationalCone() projects the input mutational catalogue onto the cone in $S$ spanned by the reference signatures with the very fast coneproj R package (https://cran.r-project.org/web/packages/coneproj). Because projections are simply calculated as scalar products, this function requires the user to specify an orthonormal basis of $S$ toghether with the components of the reference signatures with respect to it. These two input matrices can be calculated with the function SignatureSubspace() once and for all, before iterating MutationalCone() on all catalogues. SignatureSubspace() finds an orthonormal basis of $S$ with the Gram-Schmidt algorithm.

SignatureSubspace <- function(signatures){
 # signatures: (K,N)-matrix with reference signatures in columns (e.g. # COSMIC signatures)
 # with K = number of mutation types (e.g. 96), N = number of reference # signatures

 # Orthonormalization of the subspace generated by reference signatures
 S <- signatures
 S.qr <- qr(S)
 Q <- qr.Q(S.qr) # orthonormal basis of the subspace
 R <- qr.R(S.qr) # components of the reference signatures in the orthonormal basis

 return(list(Q=Q, R=R))
}

MutationalCone <- function(catalogue, Q, R){
 # catalogue: vector of length K with the mutational catalogue,
 # Q: matrix with the orthonormal basis of the subspace generated
 # by the reference signatures in columns
 # R: matrix with the components of the reference signatures wrt
 # the orthonormal basis in columns. Q and R are found with # SignatureSubspace()

 require(coneproj)

 # Projection of the catalogue onto the subspace generated by
 # reference signatures
 proj.subspace <- t(Q) %*% catalogue

 # Projection onto the cone spanned by the signatures
 weights <- as.vector(coneB(y=as.vector(proj.subspace),delta=R)$coefs)
 return(weights)
}
